# Young-Onset Diabetes in Sri Lanka: Experience From the Developing World

**DOI:** 10.1155/jdr/7557153

**Published:** 2024-12-17

**Authors:** Maulee Hiromi Arambewela, Shani A. D. Mathara Diddhenipothage, Chandrika Jayakanthi Subasinghe, Umesha Nuwanrasee Wijenayake, Surangi Jayakody, Gowri M. Ratnayake, Charles Antonypillai, Sachith Abhayaratne, Chaminda Garusinghe, Prasad Katulanda, Noel Somasundaram, Uditha Bulugahapitiya, Manilka Sumanatilleke, Achini Wijesinghe, Dimuthu Muthukuda, Sivatharshya Pathmanathan, Tharanga Samarasekara, V. T. S. Kaluarachchi, Gayani Samarasinghe, Nipun Lakshitha de Silva, Sumudu Nimali Seneviratne, Jananie Suntharesan, Sonali Sihindi Chapa Gunatilake

**Affiliations:** ^1^Department of Physiology, Faculty of Medical Sciences, University of Sri Jayewardenepura, Nugegoda, Sri Lanka; ^2^Diabetes & Endocrine Unit, National Hospital Sri Lanka, Colombo 10, Sri Lanka; ^3^Diabetes & Endocrine Unit, District General Hospital Avissawella, Avissawella, Sri Lanka; ^4^Diabetes & Endocrine Unit, General Hospital Chilaw, Chilaw, Sri Lanka; ^5^Department of Community Medicine, Faculty of Medical Sciences, University of Sri Jayewardenepura, Nugegoda, Sri Lanka; ^6^Diabetes & Endocrine Unit District Hospital Mathale, District General Hospital Mathale, Mathale, Sri Lanka; ^7^Diabetes & Endocrine Unit, National Hospital Kandy, Kandy, Sri Lanka; ^8^Department of Pharmacology, Faculty of Medicine, University of Colombo, Colombo, Sri Lanka; ^9^Diabetes & Endocrine Unit, Colombo South Teaching Hospital, Dehiwala, Sri Lanka; ^10^Department of Clinical Medicine, Faculty of Medicine, University of Colombo, Colombo, Sri Lanka; ^11^Diabetes & Hormone Centre, Nawaloka Hospital, Colombo 2, Sri Lanka; ^12^Diabetes & Endocrine Unit, Provincial General Hospital Badulla, Badulla, Sri Lanka; ^13^Diabetes & Endocrine Unit, General Hospital, Sri Jayewardenepura Kotte, Sri Lanka; ^14^Diabetes & Endocrine Unit, Teaching Hospital Kalutara, Kalutara, Sri Lanka; ^15^Diabetes and Endocrine Unit, District General Hospital, Hambantota, Sri Lanka; ^16^Diabetes & Endocrine Unit, District General Hospital, Gampaha, Sri Lanka; ^17^Department of Clinical Sciences, Faculty of Medicine, General Sir John Kotelawala Defence University, Lavinia, Sri Lanka; ^18^Department of Paediatrics, Faculty of Medicine, University of Colombo, Colombo, Sri Lanka; ^19^Department of Diabetes & Endocrine, Teaching Hospital Kurunegala, Kurunegala, Sri Lanka; ^20^Diabetes & Endocrine Unit, District General Hospital, Nuwara Eliya, Sri Lanka

**Keywords:** database, developing world, resource-limited setting, Sri Lanka, Type 1 diabetes, Type 2 diabetes, young-onset diabetes

## Abstract

**Background:** Young-onset diabetes (YOD) is characterised by unique diagnostic and management challenges more pronounced in resource-limited settings like Sri Lanka.

**Aims:** We aimed to ascertain the prevalence, patterns and characteristics of YOD in Sri Lanka and describe the state of care.

**Methods:** Retrospective review of baseline data of all patients enrolled in the prospective multicentre Database for Young-Onset Diabetes, Sri Lanka (DYOD-SL), was performed, from April 2021 to April 2023.

**Results:** A total of 2531 patient data were included from 28 centres island-wide. Females were 57.6%. The median age was 20 years (interquartile range (IQR) 17, 23), and the age at diagnosis was 15 years (IQR 12, 18). Type 1 diabetes (T1D) was the commonest (57.6%), followed by Type 2 diabetes (T2D) at 34.3%. Younger age at disease onset (*p* < 0.001), lower BMI (*p* < 0.001), and diabetic ketoacidosis (DKA) at presentation (*p* < 0.001) favoured T1D. In the total cohort, the median HbA1c was 9.8% (IQR 7.8, 12.1) with younger patients having poorer control (*p* = 0.001). Prevalence of nephropathy was 8.1%, retinopathy was 6.6%, neuropathy was 4.1%, moderate–high-risk diabetic foot disease was 1.9%, and macrovascular complications were 0.5%. Hypertension and dyslipidaemia occurred in 2.7% and 14%, respectively. Among patients > 18 years, overweight and obese were 22.2% and 10.4%. Corresponding prevalence in the 5–18-year age group was 20% and 14.7%. Among the insulin users (76%) in the total cohort, the majority (64.7%) were on premixed-based insulin regimens delivered by syringes. Self-monitoring of blood glucose (BG) was reported in 71.3% of the total population. None were on continuous/flash glucose monitoring or insulin pumps.

**Conclusion:** T1D was the commonest subtype of YOD in this hospital-based population. However, T2D was notably higher and is of significant concern. Overall, suboptimal glycaemic control and high rate of complications were noted along with substandard insulin regimens and BG monitoring.

## 1. Introduction

Young-onset diabetes (YOD) is a global epidemic, that raises concerns to individual health, and wellbeing as well as to economic productivity of countries from lost years of healthy life. The exact estimates of the burden of YOD are limited worldwide. There is sparsity of data specifically from low- and middle-income countries such as Sri Lanka, who are facing the fastest growth of the epidemic of diabetes.

Type 1 diabetes (T1D) is the commonest cause of diabetes among children and adolescents in majority of countries worldwide [1]. However, other forms such as Type 2 diabetes (T2D), monogenic forms of diabetes (MODY), and Type 3c diabetes (T3cD) also occur in this group. The past 20 years witnessed a dramatic increase in T2D among adolescents attributed to the upsurge in obesity [2]. Although T2D occurs in all ethnic groups, recent studies highlight this growing problem, especially in low-middle income countries with greater disease burden among women < 30 years [3, 4].

There has been an annual rise in the incidence of T1D of 2%–5% worldwide [5]. The SEARCH for Diabetes in Youth study in the United States found a 21.1% rise in the prevalence of T1D from 2001 to 2009 in youth < 19 years, observed across all sex, age, and ethnic subgroups [6]. The incidence of T1D has been increasing in Asia as well [7, 8]. According to International Diabetes Federation (IDF) statistics in 2021, India has the highest estimated number of patients with T1D < 20 years (229,400) followed by the United States (157,000) [1].

In Sri Lanka, recent studies have estimated a crude prevalence of diabetes in adults of 23.0% (95% confidence interval (CI) 21.2%–24.7%) with an overall estimation of dysglycemia prevalence of 53%, in 2019 [9]. However, the prevalence data of YOD in Sri Lanka is virtually nonexistent. Many studies have highlighted the growing problem of childhood obesity, a precursor for T2D in young [10, 11]. The IDF estimates an annual incidence of T1D in Sri Lanka of 3/100,000 children < 15 years old and a prevalence of 18.2/100,000 [8]. However, this is based on only one study by S. Kalra, B. Kalra, and Sharma from Karnal in India [12]. The T1D index model for Sri Lanka estimates that there are 3740–4036 patients with T1D under the age of 20 years and that the remaining life expectancy of patients diagnosed at 10 years of age is 29 years [13].

YOD poses a unique set of diagnostic and management challenges. In a resource-poor setting such as Sri Lanka, with increasing prevalence of diabetes and young onset obesity, classifying diabetes purely based on clinical phenotype without access to biochemical investigations such as c-peptide, antibodies, and genetic screening can be challenging. Duration of diabetes is a major contributor to diabetes complications, and therefore, people developing diabetes during their youth have a higher likelihood of developing chronic complications. One in 3 youth with T1D and almost 3 in 4 youth with T2D develop at least one diabetic complication in the prime of their lives [14]. Many are the challenges encountered by low and low–middle-income countries in managing intensive diseases such as T1D. Though many technological advances have taken place in the development of better insulins, delivery devices, and glucose monitoring methods, these have not translated to improvement of care among patients in resource-limited settings such as ours due to prohibitive costs and limited availability. Thus, there is an urgent need for more cost-effective care strategies.

National-level diabetes databases/registries have been recognized as important tools in managing such chronic diseases improving the quality of care and long-term outcomes enabling systematic treatment, interventions, and follow-up [15]. World Health Organization's (WHO) DIAMOND project, EURODIAB study, and national registries such as SEARCH for Diabetes in Youth study in the United States are some such database systems/registries that have contributed to current literature on YOD, especially T1D [6, 7, 16]. However, such databases/registries are scarce in South Asia. The Indian Council of Medical Research has established a national registry of youth-onset diabetes (YDR) in the year 2000 [17]. Malaysia launched an ongoing real-time register of patients developing diabetes < 20 years old via the e-DiCARE, an online registration system in 2006 [18]. In 1971, the Diabetes Association of Sri Lanka (DASL) established a single-centre insulin bank for T1D and identified 686 patients [19]. However, the majority of patients with T1D in Sri Lanka are managed in clinics in state-sector hospitals where there is no such database. Furthermore, there is no current database on young onset T2D or other forms of YOD in Sri Lanka. Therefore, a multicentre cloud-based Database for Young-Onset Diabetes, Sri Lanka (DYOD-SL), was established in 2021 by the Sri Lanka College of Endocrinologists (SLCE) to ascertain prevalence patterns, characteristics, and state of care.

## 2. Materials and Methods

### 2.1. Study Design, Setting, and Participants

A retrospective, multicentre observational study was conducted using baseline data of all the patients enrolled in the DYOD-SL, from April 2021 to April 2023.

### 2.2. DYOD-SL

DYOD-SL is a multicentre cloud-based prospective database that was designed to estimate disease burden, streamline resource allocation/distribution, develop care strategies, and create a platform for research in YOD in Sri Lanka. Island-wide hospital-based state-funded and private diabetes clinics supervised by adult and paediatric endocrinologists were invited to enrol all patients with disease onset on or before the age of 25 years in the database. Patients with diabetes mellitus diagnosed with criteria defined by the American Diabetes Association (ADA) were included, whilst patients with gestational diabetes were excluded.

### 2.3. Description of Data

Pseudonymised data retrieved from patient medical records (PMRs) were entered into a structured cloud-based online database by the trained medical staff of relevant regional clinics (RCs). Baseline data included sociodemographic details, anthropometry data, subtype of diabetes, diabetes duration, family history of diabetes, biochemistry related to the diagnosis (C peptide levels, antibody levels, and glutamic acid decarboxylase (GAD) antibodies), details of glycaemic status (HbA1c and glucose monitoring), details of treatment regimens associated metabolic conditions, and acute/chronic diabetic complications.

Classification of diabetes subtypes was based on clinical history and supportive basic laboratory data by trained clinicians in the RCs. Specific laboratory testing with C-peptide assessment, antibody testing, and genetic analysis were performed when available (Supporting Information (available here)). Weight and height data were obtained as recorded in PMRs which were measured by trained clinic staff following standard protocol, and body mass index (BMI) was calculated (kilograms per square meter) using standard formula. WHO Asian BMI cutoffs for public health actions were utilised for BMI classification among patients ≥ 18 years [20]. For patients between 5 and 18 years, BMI classification was based on the revised growth charts proposed by the Indian Academy of Paediatrics [21]. Diabetes-related complications were recorded by the clinicians as per standard local and international guidelines [22, 23]. Nephropathy was considered positive if either albuminuria was present or serum creatinine was above the reference range. The estimated glomerular filtration rate (eGFR) was calculated by the chronic kidney disease epidemiology collaboration (CKD–EPI) formula GFR = 141^∗^ min(Scr/*κ*, 1)^*α*∗^ max(Scr/*κ*, 1)^−1.209∗^ 0.993^Age∗^ 1.018 [if female]^∗^ 1.159 [if black]. International Clinical Diabetic Retinopathy (ICDR) classification was used to record retinopathy [24]. Neuropathy was considered positive if either loss of protective sensation (LOPS), measured by Semmes–Weinstein 10 g monofilament (2/3 loss in pressure point considered as LOPS), or vibration perception (measured by a 128-Hz tuning fork or biothesiometry) was impaired. When monofilament or tuning fork was not available, the Ipswich touch test was used. Loss of sensation in > 2 sites was considered as LOPS and positive for neuropathy. Diabetes foot disease was classified as low, moderate, and high risk based on the IWGDF 2019 update [25].

### 2.4. Statistical Analysis

All data percentages were given from available recorded data, and missing data was removed from the analysis. The distribution of continuous variables was tested with the Shapiro–Wilk test, and linearity was established by visual inspection of a scatterplot. Categorical variables were expressed as frequencies and percentages, whilst continuous variables were reported as median and interquartile range (IQR) (25th–75th percentile). The significance of the differences between proportions (%) and medians was tested using chi-square and/or Fisher's exact test for categorical variables and the Mann–Whitney *U* test for continuous variables. Binomial regression was performed to ascertain the effects of clinical features on the likelihood of a diagnosis of T1D and T2D and the occurrence of common complications: nephropathy and retinopathy. Statistical significance was considered if *p* < 0.05. Statistical analyses were performed using SPSS, Version 17 (IBM).

### 2.5. Ethics

Ethical approval was obtained from the Ethics Review Committee, Faculty of Medical Sciences, University of Sri Jayewardenepura (Ref No 11/21). Institutional approval was obtained by involved centres which had individual institutional ethics committees. Broad informed consent was obtained from adult patients, whilst parental consent and assent were obtained from children and adolescents aged 12–18 years, respectively.

## 3. Results

### 3.1. Baseline Characteristics of the Total Population

**Figure 1:**
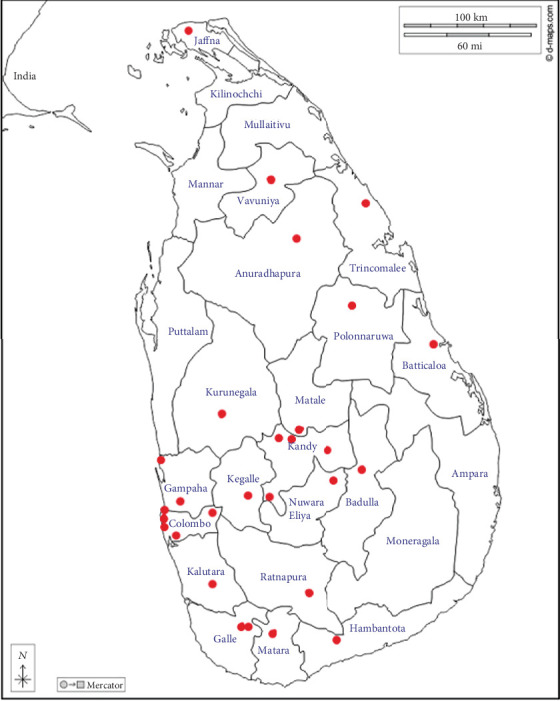
Sri Lankan map with the distribution of regional centres.

A total of 2531 records of YOD were included in the DYOD-SL database from January 2021 to November 2023, from 26 adults and two paediatric centres (Figure 1). In this study population, females were 57.6% (*n* = 1458), median age was 20 years (IQR 17, 23), median age at diagnosis was 15 years (IQR 12, 18), and the duration of diabetes was 4 years (IQR 2, 8). The majority 55% (*n* = 1392) had a positive family history of diabetes. Median BMI was 20.5 kg/m^2^ (IQR 17, 23). T1D was the commonest 57.6% (*n* = 1455) subtype recorded, followed by T2D 34.3% (*n* = 867) whilst 209 (8.1%) had other types of diabetes (Table 1). Classification of diabetes was based on clinical criteria in the vast majority 94.6% (*n* = 2394) with limited supportive biochemical testing; c-peptide assessment in 10% (*n* = 253) and antibody testing in 5% (*n* = 126).

### 3.2. Glycaemic Control, Diabetes-Related Complications, and Cardiovascular Risk Factors in the Total Population

Among patients with recorded baseline HbA1c at the time of data entry (*n* = 1531), the median HbA1c was 9.8% (IQR 7.8, 12.1). The majority 64.9% (994/1531) had a HbA1c > 8.5%. Glycemic control was significantly poorer among younger age groups in the total population and among subtypes (*p* = 0.001).

Among those screened, microvascular complications were more common with nephropathy in 8.1% (110/1353), retinopathy in 6.6% (83/1257), neuropathy in 4.1% (56/1351), and moderate to high-risk diabetic foot in 1.7% (29/1491). Macrovascular complications were reported in 0.5% (9/1781). Prevalence of hypertension was 2.7% (57/2105) and dyslipidaemia 14.1% (288/2045), whilst overweight and obesity were 22.2% and 10.4%, respectively, among adults (*n* = 1479) and 20% and 14.7%, respectively, in the 5–18-year age group (*n* = 712). Apart from dyslipidaemia being significantly higher among females (*p* = 0.027), there was no other significant gender difference in glycemic control, complications/comorbidities.

### 3.3. Diabetes Management of the Total Population

Of the total cohort, the majority 56% (1292/2313) were on insulin only, whilst 24% (554/2313) were on oral medication only. Rest was on both modes of therapy. Premixed insulin-based regimens were the most popular with 64.7% (1101/1701) of insulin users on these regimens. Syringes were the main mode of insulin delivery. Regarding blood glucose (BG) monitoring, 71.3% (1205/1691) practiced self-capillary blood glucose monitoring (SMBG), whilst 28.7% (486/1691) relied only on periodic lab reports. None engaged in continuous glucose monitoring and insulin pump therapy.

### 3.4. Clinical Characteristics and Management of Diabetes Subtypes

#### 3.4.1. T1D

T1D was the commonest subtype (57.6%, *n* = 1455) out of which females were 56.4%. Age at diagnosis was 14 years (IQR 10, 17), and females were younger (13 years vs. 14 years *p* = 0.001). Median BMI was 18 kg/m^2^ (IQR 16, 21) with females having a significantly higher BMI (19 kg/m^2^ vs. males 18 kg/m^2^, *p* < 0.001). Overweight and obesity among patients ≥ 18 years were 14.7% (124/843) and 3.2% (27/843), whilst in the 5–18-year age group, they were 10% (42/427) and 5% (21/427, respectively.

Among those who had an evaluation with C-peptide levels (*n* = 296), 64.2% had values < 0.6 ng/mL. Among those who underwent anti-GAD antibody analysis within 2 years of diagnosis, 74% (74/100) were positive. Premixed insulin-based regimen was the commonest insulin regimen used by 61% (796/1296), whilst flexible multidose insulin (MDI) was reported only in 15.3% (198/1296). Glycemic monitoring was via SMBG in 79%, whilst 21% relied only on lab reports. None were on continuous/flash glucose monitoring or on insulin pump therapy. Overall HbA1c was 10% (IQR 8, 13) with 71% (757/905) having suboptimal BG control of HbA1c > 8.5%. Among the entire cohort of T1D, diabetes ketoacidosis (DKA) was experienced by 39.8% (509/905), out of which 61.4% (313/509) reported DKA only at diagnosis and 8% (47/509) reported > 1 episodes of DKA per year. Severe hypoglycemia was experienced by 14% (156/1104), and females reported significantly higher episodes of severe hypoglycaemia (2) (1, 3) than males (1) (0, 2), *p* = 0.002 since diagnosis. Disease-related complications and cardiovascular risk factors are shown in Table 2.

#### 3.4.2. T2D

A third of the cohort (34.3%, *n* = 867) had T2D among which 59.9% were females. Median age at diagnosis was 16 years (IQR 13, 20). Median BMI was 23 kg/m^2^ (IQR 21, 27). There was no significant difference in BMI between genders. High prevalence of overweight 34.6% (176/509) and obesity 23% (117/509) was noted among adults (age ≥ 18 years), as well as in children (age 5–18 years): overweight 40% (287/719) and obesity 33%(239/719). Of the total T2D cohort, the majority (61%) was on oral medication only, whilst 39% (302/767) were on insulin with or without oral medication. HbA1c was 9% (IQR 7, 12) with 54% having HbA1c > 8.5%. SMBG was practiced only among 55% of the T2D cohort. Disease-related complications and cardiovascular risk factors are shown in Table 2. Among these, only nephropathy was significantly higher among females than males (*p* = 0.03). DKA was reported in 9.2% (65/708) which is likely to reflect ketosis-prone T2D. Among these patients who developed DKA, 6/65 had GAD antibodies done, and all were negative, whilst 50% (33/65) had C-peptide done. The median values were 2.5 ng/mL (IQR 1.1, 4.8).

##### 3.4.2.1. T1D vs. T2D

Glycemic control was significantly poorer with high HbA1c (*p* < 0.001), and disease-related complications such as severe hypoglycemia (*p* < 0.001), DKA (*p* < 0.001), and retinopathy (*p* = 0.001) were significantly higher among T1D population.

#### 3.4.3. Other Types of Diabetes

Other types of diabetes were seen among 8.1% (209/2531) of the population among which 37.7% (79/209) had pancreatic disease. Among the 79 patients with a pancreatic disease, 67 had thalassaemia, 10 had chronic pancreatitis, 1 had pancreatic resection, and 1 had hemochromatosis. Clinical features favouring MODY were seen among 66 patients; however, only one patient had genetic confirmation. The rest of the patients had diabetes related to congenital syndromes or medication.

### 3.5. Regression Analysis

Binomial regression analysis was performed to predict the diabetes subtype with the traditional clinical parameters such as age of disease onset, family history, BMI, and ketoacidosis at presentation as independent variables. Younger age at diagnosis (*B* = 1.101, *p* < 0.001), lower BMI (*B* = 1.246, *p* < 0.001), DKA at presentation (0.225, *p* < 0.001) were useful in predicting T1D diagnosis, whilst higher age at diagnosis (*B* = 0.917, *p* < 0.001) and higher BMI (*B* = 0.776, *p* < 0.001) were useful in predicting T2D diagnosis. Family history of diabetes was not a useful factor in differentiating the type of diabetes.

Regarding microvascular complications, higher duration of diabetes could consistently predict the occurrence of nephropathy in T1D (*B* = 0.887, *p* < 0.001) and T2D (*B* = 0.757, *p* < 0.001), whilst higher HbA1c in T1D predicted nephropathy (*B* = 0.848, *p* < 0.004) and retinopathy (*B* = 0.830, *p* < 0.002) occurrence. Additionally, age was positively associated with retinopathy diagnosis in T1D (*B* = 0.870, *p* < 0.001).

## 4. Discussion

Our study represents one of the largest studies in South Asia to examine the prevalence of diabetes subtypes, clinical characteristics, and status of care in YOD.

T1D was the commonest (57.6%) subtype of diabetes observed in our cohort, which is in accordance with published data from large multicentre observational studies such as 54.5% in SEARCH for Diabetes in Youth study (SEARCH) in the United States (2001–2009), 63.9% in the YDR in India (2000–2011), and 69.2% in the e-DiCARE in Malaysia (2006–2007) [Ref 7–9]. Despite the significant regional heterogeneity, the incidence of T1D is increasing worldwide [10–12]. One-fifth of the entire worldwide population of T1D is residing in low-income and lower middle-income countries. [13]. Neighbouring India is one of the 10 countries with the highest prevalence of T1D and is estimated to house 229,400 patients with T1D under the age of 20 years in 2021, according to the IDF [1]. Our study included 1455 (*n* = 850, age < 20 years) patients with T1D from 2021 to 2023, which is considerably less than what is predicted by Gregory et al., for Sri Lanka in their modelling study. They estimated 3877 (95% uncertainty interval (UI) (3740–4036) patients aged < 20 years and 9665 (UI 9162–10,143) patients aged > 20 years, for the year 2021, living with T1D in SL [13]. This observed discrepancy in prevalence is likely multifactorial: (i) Prevalence estimations were based on regional T1D incidence and disease-related mortality as Sri Lanka lacks specific data [15]. However, Sri Lanka owns a unique free health care system with island-wide coverage that is notably different to the region; (ii) DYOD-SL database included only endocrinologists who lead diabetes clinics, and there could be a significant number of patients still followed up in other states/private sector general medical/paediatric clinics and not represented in the database; (iii) high disease-specific mortality due to acute complications could preclude diagnosed/nondiagnosed T1D patients getting enrolled into the database. However, with the establishment of the DYOD-SL database and the stepping in of nongovernmental organizations to provide essential resources to patients, there is rising awareness noted within the health care system, and an increasing number of patients are currently being referred to these regional centres for care and follow up. Hence, we believe that this will pave the way for future evaluation with a larger and wider group of patients.

Furthermore, we observed a higher prevalence of T2D in our cohort (1/3^rd^) when compared to previously reported studies (15.9% in the SEARCH database, 25.3% in YDR, and 17.5% in DiCARE) [6, 17, 18]. As the published observational data are more than a decade old, this increased prevalence might reflect the increasing epidemic of T2D across all age groups regionally and globally. Obesity is one of the key mediators in the pathogenesis of T2D, and it is the epidemic of obesity that drives and fuels the global epidemic of T2D. In our cohort of young onset T2D, 57% of patients aged ≥ 18 years and 73% of patients in the 5–18-year age group were either overweight or obese. Several local studies have highlighted the high prevalence of obesity among younger people with and without diabetes highlighting the concern of obesity among younger age groups [10, 11, 26]. Given this background and the aforementioned limitations of the DYOD-SL database, a significant proportion of patients diagnosed/undiagnosed with less severe disease phenotypes could be precluded from our hospital-based study due to selection bias. Therefore, the burden of young onset T2D observed in this study could well be the tip of the iceberg. Furthermore, the prevalence of insulin therapy among T2D despite low disease duration implicating early beta cell failure as well as the occurrence of ketosis-prone T2D diabetes, which contrasted with the usual obese male phenotype, indicates overlaps with entities such as “thin fat” phenotypes, double diabetes, as highlighted in other countries in the region bearing evidence towards the enigmatic pathophysiology of YOD [27–29].

Apart from T1D and T2D, T3cD MODY is another aetiology of YOD. Recent studies show that among the 22 million population in Sri Lanka, an estimated 2000 people have severe thalassaemia [30]. In our study, 67 out of 79 YOD with associated pancreatic disease had thalassaemia. However, this was purely based on clinical criteria and warrants further biochemical and imaging studies to determine the exact aetiological nature and preventive strategies. Furthermore, the development of a region-specific MODY calculator would be beneficial in the classification of this important entity in a more pragmatic approach when confirmatory genetic testing is largely prohibitive.

Our study emphasised the utility of proper clinical assessment at presentation for meaningful classification of diabetes to plan appropriate care in resource-limited settings like Sri Lanka. Our findings indicate that age at disease onset, BMI, and DKA at presentation proved to be reliable in classifying between T1D and T2D. However, we noted that family history was not a very useful information in clinical classifications of diabetes. This is very likely due to the significantly high prevalence of T2D in the background population.

Achieving optimal glycaemic control is more challenging in YOD in comparison to adults due to inherent concerns of dynamic physical, behavioural, and psychosocial needs in this group of patients. This was reflective in our population as well. We observed suboptimal glycaemic control, across the cohort (HbA1c > 8.5% in 65%), as well as in subgroups (HbA1c > 8.5% in 71% of T1D, 54% of T2D). SEARCH data reported HbA1c > 9.5% in 16.8% of T1D and 26.6% in T2D. However, the proportion of patients with substandard control was notably higher in our cohort compared to published data from the developed world [7, 27, 28]. Additionally, we observed a high rate of DKA and the occurrence of major and minor hypoglycaemia. Overall high prevalence of suboptimal care in our cohort is largely related to limited access to optimal insulin regimens, safe and comfortable insulin delivery methods, BG monitoring devices/consumables, and structured education. Similar challenges have been reported in other low–middle-income countries in Southeast Asia, highlighting the fact that although many technological advances have taken place in diabetes management in the developed world (smart pens and hybrid close loop systems), these have not translated to improvement of care among patients in resource-limited settings such as ours due to their prohibitive costs and limited availability [31, 32].

Despite a short disease duration, a high prevalence of diabetes-related complications was reported across the study population. However, a notable proportion did not receive screening. There is consistent evidence globally as well as regionally to support that T2D in youth and adolescents is associated with a higher burden of complications within a relatively short disease duration [33]. However, in our study, all acute complications as well as retinopathy were significantly higher among the T1D population. These statistics along with the modelling statistics of T1D by Greogry et al. point towards the high morbidity and mortality of T1D in Sri Lanka reflective of the substandard monitoring and treatment regimens. Recent initiations such as the StEP-D project of the SLCE have focused on resource development/distribution and training of trainers' sessions for health care workers throughout these regional centres aiming to improve the quality of care and empower people living with T1D. These measures have been strengthened by organizations such as Life for a Child, offering much needed ongoing support and resources potentially paving the way for a change in the landscape of T1D in Sri Lanka. Nevertheless, the authors are also acutely aware of the looming threat of young onset T2D which is very likely to be just the tip of the iceberg requiring a much wider approach with multiple stakeholders focusing on healthy living to curtail its rapid inclination.

### 4.1. Strengths and Limitations

Our study was based on the largest nationwide database of YOD in the country providing a good coverage and insight into the YOD profile in Sri Lanka. Robust patient identification and systematic data entry were noted as strengths.

A significant limitation is that the subtyping of diabetes was based mainly on clinical criteria reflecting real-world nature in a resource-limited setting, and thus, potential misclassifications would be unavoidable. Another limitation is that this is predominantly a state-sector, hospital-based database involving endocrinologist-led clinics, thus underrepresenting medical/paediatric clinics in the state and private sector hospitals. Wider coverage is required to gain better insight into the true prevalence as it is likely that many patients especially those with T2D are not being captured in our data.

## 5. Conclusions

T1D was the commonest subtype of diabetes observed in the YOD population in Sri Lanka in accordance with published observational studies. However, the prevalence of T2D in the study was notably higher than reported and is of significant concern. Overall, suboptimal BG control was noted across the cohort as well as in subgroups, with high rate of acute and chronic diabetes complications despite the short duration of diabetes. Urgent attention is required for resource allocation and strategy development to optimise diagnostic accuracy, insulin regimens, BG monitoring, and health education to alleviate morbidity and mortality of this challenging disease entity.

### 5.1. Recommendations for Regional Low–Middle-Income Countries

• Establishing prospective databases/registries in YOD in developing countries to ascertain disease burden, study disease patterns, and plan out care strategies.
• Recognize YOD as an important entity requiring special attention.
• Improve care strategies in T1D through governmental and nongovernmental organizations.
• State and private sector partnerships to create public health awareness on curtailing young onset obesity and T2D.

## Figures and Tables

**Table 1:** Distribution of subtypes of diabetes.

**Subtypes**	**Total (** **n** = 2531**)**	**Females (57.6%) (** **n** = 1458**)**	**Males (42.4%) (** **n** = 1073**)**	**p** ** value**
Type 1 diabetes	1455 (57.6%)	820 (56.4%)	635 (43.6%)	0.129
Type 2 diabetes	867 (34.3%)	519 (59.9%)	348 (40.1%)	0.094
Other types	209 (8.1%)	119 (56.9%)	90 (43.1%)	0.196

*Note:p* values; males versus females.

**Table 2:** Summary of clinical characteristics of the overall population and the two commonest diabetes subtypes.

	**Total (** **n** = 2531**)**	**T1D (57.6%) (** **n** = 1455**)**	**T2D (34.3%) (** **n** = 867**)**
Current age	20 (17, 23)	20 (16, 23)	20 (17, 23)
Age at diagnosis	15 (12, 18)	14 (10, 17)	16 (13, 20)
Duration of diabetes	4 (2, 8)	5 (2, 10)	3 (1, 5)
Family history of diabetes	55% (1209/2280)	43.6% (563/1289)	73.7% (536/727)
HbA1c level (%)	9.8% (7.8, 12.1)	10 (8, 13)	9 (7, 12)
Patients with HbA1c < 6.5%	10.6% (163/1531)	6.1% (55/905)	18.4% (95/510)
HbA1c 6.5%–8.5%	24.4% (374/1531)	10.4% (93/905)	13.6% (70/510)
HbA1c > 8.5%	64.9% (994/1531)	83.6% (757/905)	68.8% (351/510)
Occurrence of DKA	27.4% (593/2165)	39.8% (509/1279)	9.2% (65/708)
Occurrence of severe hypoglycemia	10.1% (170/1679)	14.1% (156/1104)	4.4% (24/536)
Median number of severe hypoglycemia	1 (1, 2)	1 (1, 3)	1 (1, 1.2)
Macrovascular complications^a^	0.5% (9/1781)	0.6% (6/1050)	0.3% (2/577)
Retinopathy	6.6% (83/1257)	8.0% (64/800)	3.3% (12/360)
Nephropathy	8.1% (110/1353)	8.3% (70/842)	6.8% (29/428)
Neuropathy	4.1% (56/1351)	4.7% (39/828)	3.1% (13/423)
Mod-high-risk diabetic foot	1.9% (29/1491)	2.2% (20/895)	1% (5/493)
Hypertension	2.7% (57/2105)	2.5% (31/1242)	3.5% (24/680)
Dyslipidemia	14.1% (288/2045)	14.3% (172/1206)	14.7% (98/668)
BMI (kg/m^2^) (total population)	20.5 (17, 23)	18.6 (16, 21l)	23.9 (21, 27)
BMI categories > 18 year (*n* = 1479)			
Underweight (< 18.5 kg/m^2^)	26.4% (391/1479)	36.5% (308/843)	58.8% (45/509)
Normal weight (18.5–23 kg/m^2^)	40.9% (605/1479)	45.6% (385/843)	33.5% (171/509)
Overweight (23–27.5 kg/m^2^)	22.2% (329/1479)	14.7% (124/843)	34.5% (176/509)
Obese (≥ 27.5 kg/m^2^)	10.4% (154/1479)	3.2% (27/843)	23.1% (118/509)

*Note:* BMI and systolic and diastolic blood pressure reported only for patients ≥ 18 years.

Abbreviations: BMI, body mass index; DKA, diabetes ketoacidosis; T1D, Type 1 diabetes; T2D, Type 2 diabetes; T3cD, Type 3c diabetes.

^a^Composite of ischaemic heart disease, cerebrovascular disease, and peripheral vascular disease.

## Data Availability

The data that support the findings of this study are available on request from the corresponding author. The data are not publicly available due to privacy or ethical restrictions.

## Supporting Information

Supporting Information Additional supporting information can be found online in the Supporting Information section. The classification system used in this study for subtyping of young-onset diabetes is available as supporting information.
