# Supplementary material for: Young-Onset Diabetes in Sri Lanka: Experience From the Developing World
Source: J Diabetes Res. 2024 Dec 17;2024:7557153. doi: 10.1155/jdr/7557153 (PMC11668545; doi:10.1155/jdr/7557153)
Supplement: Supporting Information — Additional supporting information can be found online in the Supporting Information section. The classification system used in this study for subtyping of young-onset diabetes is available as supporting information. [file 7557153.f1.docx]

***Supplementary Material***

**Classification of diabetes**

**Clinical T1DM** – Most discriminative features are young onset diabetes with low BMI <25Kgm^2^ , unintentional weight loss, keto acidosis (DKA) and glucose levels >20 mmol/l (>360 mg/dl) at presentation [1].

**Confirmed T1DM -** Presence of clinical features of T1DM and antibody positivity or random C peptide (performed within 5 hours of eating ) <0.2 nmol/l (0.6 ng/ml) when blood glucose is at least >140 mg/dl [1].

**T2DM -** Presence of obesity, acanthosis or other features of metabolic syndrome, strong family history, absent autoimmunity, absence of insulin dependence and past history of gestational diabetes mellitus in females.

**T3cDM** – Pancreatic pathology leading to diabetes (eg- haemochromatosis, thalassemia, chronic pancreatitis, fibro-calculous pancreatic diabetes, cystic fibrosis) evident by radiological features of pancreatic pathology and C peptide <0.2 nmol/l (0.6 ng/ml) when blood glucose >140 mg/dl.

**Ketosis prone type 2 diabetes -** Occurrence of ketosis and presence of obesity, and family history suggestive of T2DM and absence of typical features of T1DM. Does not require long term insulin therapy.

**Clinically maturity onset diabetes of young (MODY) -** Positive family history for 3 generations with autosomal dominant mode of inheritance, absence of clinical and biochemical features of T1DM or features of metabolic syndrome and **n**ot requiring insulin for glycemic control for 5 years after diagnosis of diabetes.

**Confirmed maturity onset diabetes of young (MODY) -** Clinical features of MODY and positive genetic studies.

**Endocrinopathy-related diabetes** – Diabetes associated with Cushing's syndrome, acromegaly/gigantism, phaeochromocytoma, hyperthyroidism, glucagonoma and somatostatinoma

**Drug induced diabetes** – Diabetes due to steroids, antipsychotics, antiretroviral drugs, cyclosporin / tacrolimus which cause toxicity to β cells.

**Syndrome related diabetes** – Diabetes occurring with syndromes such as Down's, Klinefelter, Prader willi etc.

**Other** – Any other cause of diabetes not fitting in with any of above.
